# State of the Art: Lung Cancer Staging Using Updated Imaging Modalities

**DOI:** 10.3390/bioengineering9100493

**Published:** 2022-09-22

**Authors:** Nihal M. Batouty, Gehad A. Saleh, Ahmed Sharafeldeen, Heba Kandil, Ali Mahmoud, Ahmed Shalaby, Maha Yaghi, Adel Khelifi, Mohammed Ghazal, Ayman El-Baz

**Affiliations:** 1 Department of Diagnostic and Interventional Radiology, Faculty of Medicine, Mansoura University, Mansoura 35516, Egypt; 2 Bioengineering Department, University of Louisville, Louisville, KY 40292, USA; 3 Information Technology Department, Faculty of Computers and Informatics, Mansoura University, Mansoura 35516, Egypt; 4 Electrical, Computer, and Biomedical Engineering Department, Abu Dhabi University, Abu Dhabi 59911, United Arab Emirates; 5 Computer Science and Information Technology Department, Abu Dhabi University, Abu Dhabi 59911, United Arab Emirates

**Keywords:** chest, lung cancer, diffusion-weighted MRI, computed tomography (CT), FDG PET/CT

## Abstract

Lung cancer is among the most common mortality causes worldwide. This scientific article is a comprehensive review of current knowledge regarding screening, subtyping, imaging, staging, and management of treatment response for lung cancer. The traditional imaging modality for screening and initial lung cancer diagnosis is computed tomography (CT). Recently, a dual-energy CT was proven to enhance the categorization of variable pulmonary lesions. The National Comprehensive Cancer Network (NCCN) recommends usage of fluorodeoxyglucose positron emission tomography (FDG PET) in concert with CT to properly stage lung cancer and to prevent fruitless thoracotomies. Diffusion MR is an alternative to FDG PET/CT that is radiation-free and has a comparable diagnostic performance. For response evaluation after treatment, FDG PET/CT is a potent modality which predicts survival better than CT. Updated knowledge of lung cancer genomic abnormalities and treatment regimens helps to improve the radiologists’ skills. Incorporating the radiologic experience is crucial for precise diagnosis, therapy planning, and surveillance of lung cancer.

## 1. Introduction

Lung cancer is among the most frequent mortality causes worldwide [1]. Patients suffering from lung cancer are first diagnosed by detecting a solitary pulmonary nodule (SPN) or a pulmonary mass by computed tomography (CT) or chest radiography at an early stage of the disease. Pulmonary nodules are widespread medical challenges ranging from metastasis and lung cancer to infections and other benign lesions for which management can be complex [2]. SPNs have a 30–40% chance of being malignant [3]. Precise categorization of SPNs is clinically crucial because the early diagnosis of malignant disease would increase the chances of a successful surgical resection and a 5-year survival rate [4].

The Lung Imaging Reporting and Data System (Lung-RADS) was first published by the American College of Radiology (ACR) in 2014 to enable standardized reporting and management of abnormal findings in lung cancer screening [5,6].

The ACR updated the Lung-RADS version 1.1 in May 2019, with the two main changes concerning perifissural nodules and ground-glass nodules as a negative screening result [7,8]. Lung-RADS version 1.1 is better at categorizing the solid pulmonary nodules and reduces the percentage of false positives by downgrading perifissural nodules in the range of 6–10 mm to benign lesions [8]. The Fleischner Society recommendations for the management of incidental SPNs were initially released in 2005, then further distinct guidelines for subsolid nodules were announced in 2013 [9,10].

In 2017, the latest guidelines for solid and subsolid nodules were collected in a simple table, while special recommendations were incorporated for multiple nodules. The minimum threshold size for solid nodules on routine follow-up examination was increased. Additionally, fewer follow-up assessments were proposed for stable nodules. Furthermore, a longer period was advised in the case of subsolid nodules before the primary follow-up. In addition, the follow-up period was increased to 5 years. The previous recommendations do not apply to immunosuppressed patients, lung cancer screening, or primary cancer patients [11].

This comprehensive review aims to discuss the current knowledge regarding screening, subtyping, imaging, staging, and management of treatment response for lung cancer. The updated knowledge of lung cancer genomic abnormalities and treatment protocols is now considered crucial for radiologists for precise diagnosis and as a part of a multidisciplinary team for proper management and therapy planning for lung cancer patients.

The rest of this paper is organized as follows: Section 2 discusses the classification of lung cancer. Section 3 demonstrates lung cancer screening. Section 4 illustrates TNM lung cancer staging. Section 5 presents lung cancer follow-up and response evaluation. Section 6 shows World Health Organization (WHO) criteria and Response Evaluation Criteria in Solid Tumors (RECIST). Section 7 demonstrates advances in lung cancer tumor genomics and precision therapy. Finally, Section 8 summarizes the paper.

## 2. Classification of Lung Cancer

### 2.1. Anatomic Classification

Lung cancer has two anatomic types, central and peripheral [12]. Central lung cancer commonly involves squamous cell carcinoma and small-cell carcinoma. Peripheral lung cancer commonly involves adenocarcinoma as well as large-cell carcinoma.

### 2.2. Histopathologic Classification

Following the 2021 World Health Organization (WHO) classification of thoracic tumors (which is similar to the 2015 WHO classification) [13], malignant lung tumors are classified via histopathology into (1) small-cell lung cancer (SCLC), which comprises 15–20% of malignant lung tumors, including small-cell carcinoma, (2) non-small-cell lung cancer (NSCLC), comprising 80–85% of malignant lung tumors, and (3) other, rare types of malignant lung tumors such as sarcoma and lymphoma.

The histopathologic classification of lung cancers helps in deciding treatment and prediction of prognosis [12]. NSCLC represents 85% of lung cancer patients, including squamous cell carcinoma (SCC) (40%), adenocarcinoma (ADC) (50%), and large-cell lung cancer (10%), each of which has a different prognosis [14,15]. Therefore, it is valuable to precisely differentiate between the subtypes of NSCLC before starting treatment [16].

#### 2.2.1. Small-Cell Lung Cancer (SCLC)

The SCLC/small-cell carcinoma is developed from submucosal neuroendocrine cells and is strongly linked to cigarette smoking. This type of cancer is considered the most aggressive type of primary malignant tumor of the lung, with high rate of extrathoracic metastases, which frequently exist at time of the diagnosis [17]. This type of lung cancer is usually linked to massive mediastinal and hilar lymphadenopathy and peripheral lung collapse. Superior vena cava obstruction or infiltration may occur. SCLC is chemotherapy-sensitive and might be related to paraneoplastic syndrome [12,18,19]. It has a central location in 90% of patients, and appears on X-ray as hilar or para-hilar shadow with wide mediastinum. CT appearance may simulate that of lymphoma due to massive mediastinal lymphadenopathy, but in SCLC, central necrosis of nodes is common [12].

#### 2.2.2. Squamous Cell Carcinoma

The squamous cell carcinoma is derived from the bronchial epithelium and is related to cigarette smoking. It shows the slowest growth rate among malignant lung tumors. Squamous cell carcinoma has a central location and is associated with frequent peripheral lobar or segmental collapse. Cavitation with thick irregular wall is common in 82% of patients. It is the most widespread histopathologic category of superior sulcus tumor (Pancoast tumor) [12,19].

#### 2.2.3. Adenocarcinoma

**Figure 1:**
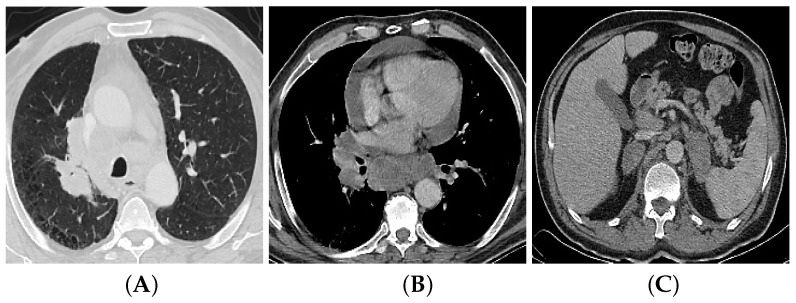
A 64-year-old male cigarette smoker with bronchogenic adenocarcinoma. Axial CT image (**A**) reveals spiculated right upper lung lobe mass (4.5×2.5 cm). Axial CT image at distal level (**B**) reveals ipsilateral hilar, mediastinal LN with central necrosis, and pericardial effusion. Axial CT image of upper abdomen (**C**) reveals bilateral metastatic suprarenal masses.

The adenocarcinoma type of lung cancer, derived from bronchial submucosal glands, is considered the most widespread histopathologic type of lung cancer in nonsmokers and young women [20]. It appears mostly as a pulmonary nodule and less frequently as a mass (diameter more than 3 cm) with a peripheral location in 72% of the cases (Figure 1). Cavitation is rare with this type of lung cancer. Adenocarcinoma can present in CT by ground-glass opacity surrounding a lung nodule (subsolid nodule), as multiple faint nodules, or, least likely, as consolidation in one lobe or in the whole lung [12,19]. Mediastinal and hilar nodal spread can occur, but in smaller volume than SCLC. Spread occurs in the same lung or the contralateral lung. Distant metastasis to the brain, bone, liver, and suprarenal glands is frequent [21].

#### 2.2.4. Large-Cell Carcinoma

Large-cell carcinoma is derived from atypical cells that have microscopic appearance of “large” cells, and it is strongly related to cigarette smoking. It has peripheral location in 63% of patients. In CT, it appears as large peripheral masses with early significant mediastinal and hilar nodal spread [12,19].

## 3. Lung Cancer Screening

Screening looks for signs of cancer before the onset of symptoms. This would help in detecting the disease at early stages and hence mitigate adverse events. In the following subsections, different lung cancer screening techniques are presented.

### 3.1. Digital Chest Radiography

Chest radiography is the most commonly used technique to rule out chest disease and to monitor patients after treatment. Although chest radiography has lower sensitivity of detecting smaller pulmonary lesions than CT, it is easily accessible, has low cost, and uses a low radiation dose [21,22].

Studies of lung cancer screening with conventional chest radiography found that it has a 23% sensitivity rate and a 96% specificity rate [23]. Digital chest radiography has the same specificity with twice or higher increased sensitivity [22].

Even with the advent of digital imaging, the use of chest radiography to detect lung cancer is still considered a real challenge because the diagnostic accuracy of early detection of lung cancer using the digital chest radiography is affected by the confidence and experience level of the reader [22].

Relying only on the chest radiography, 22–63% of malignant lung lesions could be underdiagnosed at a stage of disease that is detectable by CT. Even with the benefit of the advances in digital chest radiography over conventional chest radiography, this large number of false positive results and low detection rates are in support of the low-dosage CT for lung cancer screening [22].

#### 3.1.1. Digital Radiography Technique

The acquisition of digital radiography does not differ from that of conventional chest radiography. A direct detector unit is used for obtaining radiographs. Image processing is performed using nonlinear multifrequency band processing. Acquisition parameters differ according to the manufacturer recommendation. Postero-anterior projection only is commonly used in screening programs for lung cancer, even though adding lateral projection leads to better assessment of suspected lesions [22].

#### 3.1.2. Digital Radiography Scale for Lung Cancer Screening

The suspicious focal opacities found in radiographs can be classified into five levels, according to the de Hoop study: negative (level 1), irregular but probably negative (level 2), indeterminate for a lesion (level 3), probable lesion (level 4), and definite lesion (level 5). When radiography findings rated as levels 4–5 are referred for follow-up by CT, malignant lesions are detected in between 37% and 78% of cases (reader-dependent) [22].

### 3.2. Multi-Detector Computed Tomography (CT)

CT is deemed the routine imaging tool for lung cancer. CT provides the most detailed imaging information about tumor location, size, and extensions [12,24]. Lung cancer in CT appears as circumscribed pulmonary opacity, considered a mass if it measures more than 3 cm in diameter, and a nodule if less than 3 cm [24]. The tumor size is of little diagnostic value to differentiate benign from malignant lung lesions. Commonly, calcifications are criteria of benign lesions, particularly in the case of dense central or laminated calcifications. Yet, malignant lesions may show granular calcifications in 7% of carcinomas, whether they represent tumor calcifications or benign granuloma engulfed by the malignant tumor [19]. Lung cancer usually has an ill-defined, spiculated, irregular margin (Figure 1) and is less likely lobulated with a notch [25]. Unfortunately, ill-defined margins can be seen with benign tumors as well [19].

Lung cancer can present as either peripheral or central tumors. Peripheral tumors are mostly oval or rounded in shape; typical fine strands radiating from tumor margin into the lung, may be seen [17,25]. Associated lung collapse and consolidation is less common than in central tumors, but mucoid impaction or bronchocele may occur distal to tumor obstructing the airways. Air bronchogram and ground-glass opacity are less likely and can occur with broncho-alveolar carcinoma, which may also appear as multiple ill-defined nodules [17,26]. Cavitation with thick irregular wall and fluid level occurs with squamous cell carcinoma [17]. Central tumors are characterized by collapse or consolidation distal to the tumor; this peripheral collapse can show more contrast enhancement than the tumor itself [19,25].

CT assessment of chest wall invasion is unreliable [17]. Obliteration of the extrapleural fat plane or thickened pleura does not always mean chest wall invasion. Rib destruction or extension of pulmonary mass into the chest wall remain the definite signs. Apart from bony invasion, magnetic resonance imaging (MRI) is better than CT in assessment of chest wall invasion in certain conditions, such as in superior sulcus tumor (Pancoast tumor), an apical lung tumor which invades chest wall and appears as pleural thickening at the lung apex. It can invade the brachial plexus, cervical sympathetic chain, and subclavian vessels. CT can evaluate the relation between the tumor and different mediastinal structures: encasement of great vessels, superior vena cava obstruction, esophagus, and main bronchi [17,19]. Contact with mediastinal structures alone does not indicate mediastinal invasion. Visible tumor tissue within the mediastinal fat is a reliable sign of mediastinal invasion. Contact withmore than 3 cm with mediastinal structures and more than 90% encasement of the aorta are criteria of nonresectable tumors [19,25].

Contrast-enhanced CT provides information about the measured attenuation of lung tumors; it can assess the enhancement of lung tumors which can distinguish benign from malignant lung lesions [27].

CT scans are acquired with patients in the supine position during a single breath hold at the end of inspiration. The following are the imaging parameters; 120 kVp tube voltage, 100–250 mA tube current, and slice thickness and interval varying according to the type of CT scanner (5 or 6 mm in most scanners). Contrast material is injected intravenously (1.5 mL/kg of body weight, rate 3.0 mL/s) via a dual high-pressure injector, then 50 mL saline solution is injected. Images are reconstructed with section thickness 0.6 and slice interval 1 mm [28].

### 3.3. Dual-Energy Computed Tomography (DECT)

Recently, dual-energy CT (DECT) has been used to enhance characterization of lung lesions. Based on the type of the scanner, there are variable DECT techniques, such as dual-source machines, rapid kVp switch of single source, or dual-layer spectral detectors [29]. Previous studies using phantoms proved the high accuracy of iodine concentration (IC) maps, with slightly higher accurate results for rapid kVp switching and dual-layer spectral detectors [30]. There is high agreement between different vendors for both dual-energy and spectral CT machines, unlike the conventional CT attenuation densities that show less agreement [31]. Thus, IC maps have good potential for clinical routine use [32].

IC maps have several applications that depend on quantitative measurement of iodine density in the region of interest as a marker of vascularity. Accordingly, IC can be considered as a surrogate for tumor tissue angiogenesis and perfusion in different types of dual-energy CT machines. There was good to excellent agreement for both single-source DECT and dual-input CT perfusion parameters including IC when applied to assess the blood supply in patients with lung cancer (intraclass correlation coefficient (ICC) range was 0.82 to 0.99) [33]. IC maps were applied to successfully differentiate the lung cancer; quantitative analysis of iodine uptake images had an added value for the distinction among invasive and minimally invasive or noninvasive adenocarcinoma. Power of diagnosing invasive adenocarcinoma increased from 0.888 to 0.959 (*p*-value 0.029) after adding iodine uptake parameters [34]. DECT helps to differentiate benign from malignant pulmonary nodules by measurement of iodine value on IC image, with higher sensitivity (92.0% versus 72.0%), similar specificity (70.0% versus 70.0%), and higher diagnostic accuracy (82.2% versus 71.1%) than degree of enhancement measured by conventional post-contrast CT [35]. DECT studies evaluated the contrast enhancement and iodine uptake behavior of pulmonary metastases, which differs based on the primary tumor type [32,36], in addition to evaluating the response of the lung cancer treatment [37].

#### 3.3.1. Dual-Energy Computed Tomography (DECT) Technique

Contrast medium is intravenously injected using a 1.2 mL/kg standard dose, a 2–2.5 mL/s flow rate, then 30 mL saline flush at the similar flow rate. Venous phases scanning is obtained after 70 s scan delay time after the beginning of injecting the contrast material. The scanning parameters differ according to the CT scanner type. Scanner vendors provide reconstruction algorithms that could be used to reconstruct conventional and spectral basis images [32].

#### 3.3.2. Quantitative Analysis

The spectral images’ quantitative analysis is performed using the software provided by the manufacturer. The lesion volume is segmented in the axial plane with soft tissue window in virtual noncontrast images derived from spectral images. Then, it is compared to post-contrast images, resulting in tumor volume reduction. DECT studies recommended this approach to avoid underestimation of the true tumor volume by the exclusion of the peripheral voxels of the tumor to minimize volume averaging [38,39]. The software automatically provides the IC maps and the polychromatic images. IC is measured in mg/mL.

### 3.4. Positron Emission Tomography (PET/CT)

CT is considered the best modality to detect pulmonary lesions; however, it may not be exact enough to distinguish benign and malignant nodules [40,41]. Consequently, invasive procedures (e.g., lung biopsy or video-assisted thoracoscopic surgery) might be performed on patients with benign nodules simply to rule out malignancy [42].

PET/CT using the radiotracer 18F-Fluorodeoxyglucose (18F-FDG) has been verified to be a reliable noninvasive imaging modality to differentiate benign from malignant lesions [43]. The 18F-FDG PET/CT method estimates the rate of cellular glucose metabolism, thus it allows the detection of the metabolically active tissue. The malignant lesions contain metabolically active cells with greater glucose uptake compared with cells in benign tumors [3]. PET/CT combines both metabolic and morphologic information with high sensitivity and specificity in distinguishing malignant pulmonary lesions (96.8% and 77.8%, respectively) [43,44,45]. Thus, along with its deep-rooted role in staging of pulmonary tumor, PET/CT is a critical diagnostic tool principally used to distinguish between benign and malignant SPNs, decreasing the number of unnecessary invasive procedures for benign SPNs [3].

The National Comprehensive Cancer Network (NCCN) recommends the use of 18F-FDG PET/CT for the proper lung cancer staging and for preventing fruitless thoracotomies [46].

The dispersion of the 18F-FDG uptake in the lesions is not homogeneous. The lesion heterogeneity is related to the cellular proliferation, hypoxia, necrosis, and angiogenesis caused by the malignancy [47]. Therefore, measuring lesion heterogeneity is valuable to differentiate among benign and malignant pulmonary lesions. The tumor heterogeneity of 18F-FDG uptake was proposed to be characterized using PET image texture analysis [48].

#### 3.4.1. Patient Preparation

Patients are instructed to fast for 6 hours prior to the PET/CT scan to adjust the level of blood glucose of the fingertip blood sampling to <10 mmol/L just before 18F-FDG intravenous injection, because a glucose level that is greater than 200 mg/dL might alter the FDG biodistribution. The dose of 18F-FDG ranges from 350–400 MBq (9.5–11 mCi) based on the body weight of patients (3.0∼3.7 MBq/kg) with an uptake period of 1 h, wherein the patient is informed to rest calmly.

#### 3.4.2. PET/CT Scan Acquisition

During examinations, patients are comfortably placed on the PET/CT scanner for about 30 to 60 minutes (depending on the number of minutes per bed position and the field of view). After 60 min, the patient is typically imaged from the skull base to mid-thigh level. Noncontrast CT images are gained for attenuation correction and better anatomical localization.

After the imaging process, reconstruction of the CT images is carried out. The reconstructed CT images can then be used to generate an attenuation map to correct the attenuation of the PET images. Subsequently, a fusion between the corrected PET images and the CT images is performed to allow for the PET/CT images evaluation. All images are displayed in multiplanar reconstructions.

#### 3.4.3. Qualitative Image Analysis

The CT component images allow qualitative interpretation by visual analysis of the lesion morphology. Nodules with irregular/spiculated margins, without typical benign calcification (popcorn-like, laminated, central nodular, and diffuse), and showing interval growth are considered malignant [3].

#### 3.4.4. Quantitative Image Analysis

**Figure 2:**
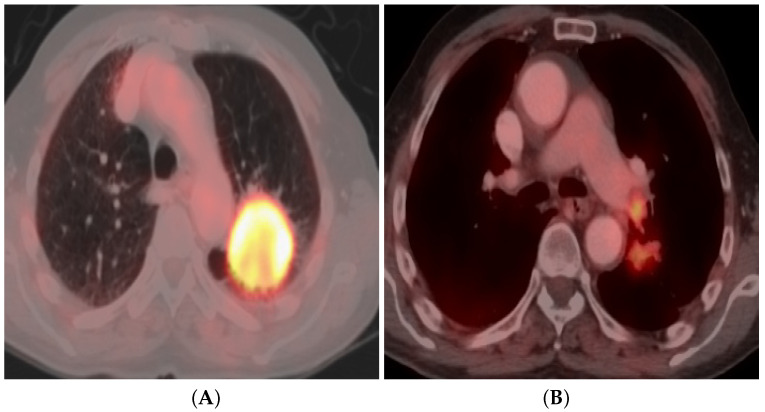
A 65-year-old male cigarette smoker with bronchogenic large-cell carcinoma. Axial-fused FDG PET/CT image (**A**) reveals FDG-avid large left upper lung lobe mass (5.5×6 cm) with maximum standardized uptake value (SUVmax) of 11.8. Axial-fused FDG PET/CT image at distal level (**B**) reveals FDG-avid ipsilateral hilar LN with SUVmax 7. No FDG-avid distant metastatic deposits, imaging-based staging IIIA (T3 N1 M0).

A standardized uptake value (SUV) is employed to semiquantitatively interpret the PET scans. SUV signifies the level of 18F-FDG uptake. If any lesion has an SUV higher than the background mediastinal blood pool uptake, then it is deemed hypermetabolic, and is expected to be malignant (Figure 2). Early studies of PET/CT in the literature approved 2.5 as a threshold SUVmax [49,50,51].

#### 3.4.5. PET/CT Imaging Pitfalls

Increased uptake of 18F-FDG can be detected in certain benign lesions with an elevated intensity of inflammatory cells, such as granulomas and bacterial or fungal infections, that could likely result in finding false positives [52,53].

In addition, the use of single-time-point (STP) PET/CT is less specific in identifying malignancy in inhabitants with endemic infectious pulmonary disease in comparison with nonendemic regions. Thus, dual-time-point (DTP) is proposed, using retention index (RI) to enhance the diagnosis specificity of the PET/CT at granuloma-endemic regions with accurate distinction of benign and malignant SPNs [4].

#### 3.4.6. Dual-Time-Point FDG PET/CT

The 18F-FDG uptake is routinely measured by semiquantitative SUVs, but some overlap in SUVs has been stated between begin and malignant nodules [54], so to overcome this diagnostic challenge, a DTP FDG-PET imaging technique can be performed to discriminate benign from malignant pulmonary lesions [55].

Malignant lesions with strong glycolysis and little or no glucose-6-phosphatase have decay-corrected SUVs on DTP imaging which progressively rise over time. This allows for improved contrast on delayed acquisition, compared to the nonincreasing SUVs of the neighboring normal tissue, due to certain glucose-6-phosphatase activity, for example, in the liver [56].

#### 3.4.7. DTP FDG PET/CT Scan Acquisition

The process starts with a low-dosage CT scan with the following scanning parameters; a 3.75 mm slice thickness, a 1.75 mm pitch, 120 keV, and auto mA (35–100 mA, according to the body mass of the patient). Immediately after that, emission scanning is conducted without requiring the patient to change positions. Examinations of the whole body take approximately 1 hour (58 min ± 5) following the injection of 18F-FDG (194.5 MBq ±27.3). In contrast, the delayed chest examinations are obtained in nearly 2 hours (120 min ± 4) following the injection of the 18F-FDG. The average time interval between early and delayed images is 60 min ± 6. The acquired attenuation-corrected CT data are then reconstructed utilizing a three-dimensional ordered-subset expectation maximization algorithm (image matrix size, 192×192; 16 subsets, 2 iterations: VUE Point Plus).

However, few of the DTP imaging studies found different sensitivity/specificity compared with STP imaging, with some positive and some negative findings [57,58]. Thus, sparse recent studies investigated the quantification of heterogeneity derived from various texture characteristics on DTP 18F-FDG PET/CT images and revealed that machine learning models trained by texture features accomplished substantial advances, compared to ordinary clinical metrics and visual assessment, in differentiating between benign and malignant SPNs, particularly by texture features on the delayed PET/CT images [4].

### 3.5. Magnetic Resonance Imaging (MRI)

MRI is of limited clinical use for lung disease patients, given that it compares poorly with other modalities such as CT, radiography, and PET/CT. Obtaining MRI of diagnostic quality of the lungs is very challenging due to many factors, including the low signal-to-noise ratio resulting from the lung’s inherent low proton density, respiratory and cardiac motion artifacts, and susceptibility artifacts at the interface between tissue and air [59]. Lung imaging quality has improved by using a combination of free breathing, breath-hold, and gated protocols [60].

Pulmonary MRI is a perfect modality for patients demanding sequential follow-up, pediatric examinations, and pregnant women owing to its lack of ionizing radiation [61]. Recent MRI techniques, such as three-dimensional gradient sequences with ultrashort echo time and zero echo time using multicoil parallel acquisitions and acceleration techniques, have improved the small lung nodule detection in MRI [62]. Additionally, recent research has revealed the potential of using MRI for lung cancer screening, compared to low-dose CT [63].

The recently published Fleischner Society position paper considered pulmonary nodule characterization and lung cancer staging as an indication for current clinical use of pulmonary MRI [61].

### 3.6. Diffusion-Weighted Imaging (DWI)

**Figure 3:**
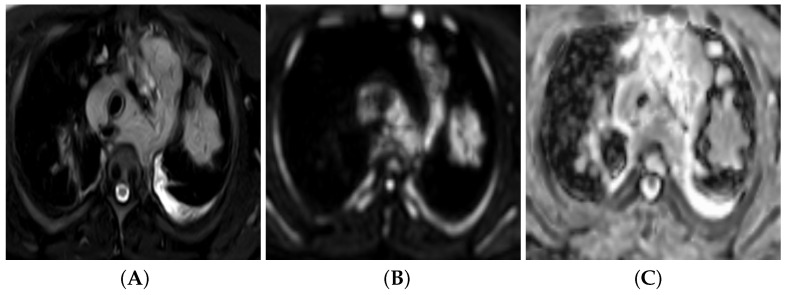
A 38-year-old male with bronchogenic adenocarcinoma. Axial T2 fast spin echo (FSE) with fat suppression (**A**) reveals spiculated left upper lung lobe mass of intermediate to high signal intensity. Diffusion-weighted image, high b-value =800 mm^2^/s (**B**) and corresponding ADC image (**C**) reveal a mass-constrained diffusion pattern with low ADC value of $1.12\times 10^{-3}$ mm^2^/s.

Diffusion-weighted imaging (DWI) was found to be the most beneficial modality for pulmonary nodule characterization compared to other MRI assessed sequences. Meta-analysis reported a sensitivity of 83% and a specificity of 80% in distinguishing benign from malignant tumors [64]. DWI is a noncontrast functional MR sequence that uses the restriction of water molecule motion to assess tissue cellularity. Malignant lung tumors reveal restricted diffusion owing to their increased cellularity and reduced extracellular space [65] (Figure 3). The apparent diffusion coefficient (ADC) map can be used to perform qualitative and quantitative DWI analysis. Malignant lung tumors have a significantly low ADC compared to benign lesions (Figure 3). Thus, DWI is considered a radiation-free alternative for characterizing pulmonary nodules [66,67].

PET/CT is very expensive, with challenging assessment in some conditions because of false-negative results for well-differentiated pulmonary adenocarcinomas and small sizes of metabolically active tumors [68], and false-positive results for inflammatory nodules [52]. A few studies comparing DWI and PET/CT to distinguish among benign and malignant pulmonary nodules found that the two imaging modalities had similar accuracies [69,70,71,72]. A recent study discussed the value of combined assessment of DWI and T2-weighted imaging (T2WI) for discrimination of lung cancer from benign pulmonary nodules and masses and revealed that this combined assessment improves the diagnostic capability [2].

Another study revealed that diagnostic performance of DWI is comparable to, or even better than, that of PET/CT for classification of pulmonary lesions as malignant or benign [42]. Meta-analysis showed that DWI can discriminate between malignant and benign pulmonary lesions [73]. In addition, DWI is useful for assessing the N factor of lung cancer, with high diagnostic potential for nodal evaluation in NSCLC [74,75].

#### 3.6.1. DW Imaging Protocol

MR images can be acquired using a 1.5 T or 3 T magnetic machine with two front coils of six-channels body-phased array and two backward spinal clusters. Conventional MR images are performed before DWI and involve an axial T1-weighted spin-echo sequence and coronal and axial T2-weighted fast spin-echo.

DWIs are acquired using a single-shot echo-planar method with a 5 mm slice thickness under spectral attenuated inversion recovery (SPAIR) with a respiratory triggered scan using diffusion gradient encoding in three orthogonal directions with variable b-values, including b-values =0 and 800 s/mm^2^ [2,67], b-values =0 and 500 s/mm^2^ [76], or b-values =20, 500, and 800 s/mm^2^ [77].

Matched ADC maps are generated using at least two images of different b-values using a workstation with functional tool software.

#### 3.6.2. Qualitative Image Analysis

Restricted diffusion is considered as visually detected hyperintense signal on DWI at high b-values of 500 and 800 s/mm^2^ and isointense or hypointense signal on the ADC map compared to adjacent, normal lung (Figure 3).

#### 3.6.3. Quantitative Image Analysis

ADC values are measured by manually applying a 2D circular or oval region of interest (ROI) centrally to the lesion on ADC map, referring to T2-weighted, occupying as much of the lesion as possible (at least 50%) without including the adjacent normal lung. The ADC values are measured three times, then the measurements are averaged.

In addition, other DWI parameters, including signal intensity (SI) score and lesion to spinal cord signal intensity ratio (LSR), are feasible in distinction between benign and malignant SPNs [76].

#### 3.6.4. Intravoxel Incoherent Motion (IVIM)

An intravoxel incoherent motion (IVIM) study is a modification of DWI protocol where images are acquired at lower than conventional b-values, which are more sensitive to blood microcirculation. The IVIM model can differentiate between lung cancer and consolidation as compared to PET–CT [78]. Three parameters can be gained from IVIM, including the true diffusion coefficient (D value), perfusion-related pseudodiffusion coefficient (D* value), and perfusion fraction (f value) [79]. Ki-67 is a nuclear antigen commonly applied as a tumor cell proliferation marker in standard pathologic analyses for lung cancer [80]. A meta-analysis showed that high Ki-67 expression is correlated with disease progression and poor outcome in lung cancer patients [81], although the assessment of Ki-67 expression using immunohistochemistry (IHC) is invasive and is often affected by tissue sample quality [82]. A recent study compared the quantitative MRI parameters from conventional DWI, IVIM, and diffusion kurtosis imaging (DKI) in correlations with tumor Ki-67 and revealed that the (D value) from IVIM displayed the best performance among the three techniques for differentiating the high-Ki-67 group from the low-Ki-67 group, as well as distinguishing SCLC from NSCLC [83].

### 3.7. Dynamic Contrast-Enhanced (DCE) MRI

The dynamic contrast-enhanced (DCE) MRI with 2D or 3D SE, turbo SE, and/or GRE sequences has been utilized for differentiating malignant from benign pulmonary nodules with a sensitivity, specificity, and accuracy ranging from 55% to 100%, 54% to 100%, and 75% to 96%, respectively [84,85,86]. Though enhancement levels may differ owing to underlying pathologic conditions such as tumor angiogenesis, the presence or absence of fibrosis, and necrosis inside the tumor, malignant pulmonary nodules display homogenous enhancement, but at variable levels on T1-weighted images following contrast media administration [84,87]. DCE MRI also provides signal intensity/time curves which represent first-transit and washout of contrast media under breath-hold or repeated breath-hold conditions for 5 min. The enhancement patterns within nodules on DCE MRI and/or parameters determined using signal intensity/time curves can be assessed to differentiate between benign and malignant nodules. Many studies indicated that enhancement patterns assessed with DCE MRI may be beneficial for the diagnosis and management of pulmonary nodules [84,85]. A meta-analysis reported no significant differences in the diagnostic performance of CECT, DCE MRI, and FDG PET in the evaluation of pulmonary nodules [88]. Consequently, DCE MRI has a corresponding role in CT and FDG PET/CT in establishing the necessity for additional intervention and treatment in routine clinical practice [87].

### 3.8. Hyperpolarized gas MRI

Lung diseases impact the airways, the gas exchange parenchyma, or both, with significant influence on patients’ morbidity [89]. Hyperpolarized (HP) gas MR imaging using helium (3He) and xenon (129Xe) offers a noninvasive, ionizing radiation–free quantitative procedure to assess pulmonary structure, gas exchange, and function [90]. Owing to the paucity in the supply of helium and its high price, the less expensive and organically available xenon has been adopted in HP gas MRI. Previous study revealed a substantial quantitative agreement for ventilation maps on hyperpolarized 3He MRI and 4DCT imaging in patients with lung cancer [91]. Additionally, HP gas MRI is helpful in radiotherapy planning for lung cancer, utilizing functional information provided by HP gas MRI that has been registered for treatment planning.CT revealed statistically significant improvements to radiotherapy planning, as well as the mean lung dose to the functional lung [92].

### 3.9. Whole-Body Magnetic Resonance Imaging (WB-MRI) and WB-DWI

Whole-body magnetic resonance imaging (WB-MRI) is a non-invasive modality for cancer staging with better diagnostic accuracy after inclusion of DWI [93]. Recent meta-analysis revealed that WB-MRI and WB-DWI are radiation-free alternatives that have diagnostic performance similar to that of 18F-FDG PET/CT in M-staging of NSCLC with reduced cost and time compared to PET/CT [94].

#### PET/MRI

Lately, hybrid PET/MRI has been involved in abdominal or pelvic cancer staging due to the opportunity to combine multiparametric metabolic, morphological, and functional data provided by radioactive tracers and various MRI sequences [95].

PET/MRI has also become popular for evaluating NSCLC and pulmonary lesions, but owing to the diagnostic challenges of the MRI lung, pulmonary nodule size is an important factor to assess PET/MRI diagnostic performance [96]. Adjusting large FOV DWI is critical for PET/MRI, especially with regard to the characterization of pulmonary nodules, and recent advances involving usage of reverse-phase encoding with DWI could assist in providing a quantitative advantage to PET/MRI [77].

Recent work analyzed the available studies on PET/MRI for staging in NSCLC or lung nodule and revealed that PET/MRI had similar sensitivity for detecting FDG-avid lung nodules and nodules greater than 10 mm, but PET/CT attained a greater detection rate in non-FDG-avid lung nodules below 5 mm. In addition, when compared to PET/CT, PET/MRI had no advantages in T- and N-staging of NSCLC [96].

## 4. Imaging and Lung Cancer TNM Staging

The latest (eighth) edition of TNM Staging System, released in January 2017, is currently followed in clinical practice for lung cancer staging [97].

### 4.1. T (Tumor) Descriptor

The scope of spread of the primary tumor is shown by the T descriptors, consisting of tumor size, invasion, and site of separate tumor in relation to the primary tumor [98]. Tumor size must be quantified with CT utilizing the lung window, and the largest dimension in any plane has to be stated [99].

The cutoff points of 3 cm, 5 cm, and 7 cm separate T1 from T2 tumors, T3 from T2, and T3 from T4, respectively. The eighth edition of lung cancer staging announced a new T category of Tis and T1mi. Tis now is allocated for carcinoma in situ of squamous cell carcinoma and adenocarcinoma, unlike the seventh edition, where Tis was only employed for squamous cell carcinoma in situ. The T descriptors are now sorted into five main categories—see Table 1.

The T classification is modified in the eighth edition of TNM lung cancer staging built on 1 cm increments in tumor size, downstage of endobronchial tumor carina to T2, integrating atelectasis or pneumonitis into T2, and considering the diaphragmatic invasion as T4 [98].

Commonly, CT well illustrates the invasion of the mediastinal structures and great vessels, and transthoracic ultrasound and MRI assess chest wall and pleural invasion better than CT [100]. For Pancoast tumors, MRI demonstrated better results than CT in preoperative staging [101].

### 4.2. N (Nodal) Descriptor

The classification of nodal metastasis is established on structural position of affected lymph nodes (LNs), unlike other cancer staging which often depend on the number of metastatic LNs. The N descriptor categorization of the seventh edition remained the same for the eighth edition, classified into four main categories (Table 1) [104].

The classic criteria for diagnosis of LN involvement is LN greater than 10 mm in short axis [102]. In the diagnosis of LN involvement, PET–CT revealed better outcomes than CT [105]. DWI can discriminate between benign and malignant LNs, as a meta-analysis revealed that diffusion MRI has the same sensitivity as PET–CT but has higher specificity than PET–CT [106], and other trials showed comparable diagnostic performance of PET–CT and DWI [107].

### 4.3. M (Metastasis) Descriptor

The M descriptors in the eighth edition for M1a intrathoracic metastasis had no significant change from the seventh edition [98]. Extrathoracic metastasis in the eighth edition was subdivided into M1b and M1c, depending on the number of metastatic lesions, due to significantly poor prognosis for those with multiple extrathoracic metastasis (M1c) (Table 1) [102].

Lung cancer frequently spreads to the adrenal glands, brain, and bone marrow [108], so an MRI provides more image contrast flexibility than CT. Cerebral staging is advised in patients with promising curative opportunities, as MRI outperforms CT in the detection of brain metastases [109]. Adrenal metastases are accurately detected by PET–CT [110]. Generally, PET–CT is the best for initial lung cancer staging compared to bone and CT scans, except for brain metastases [101].

### 4.4. Overall Stage Grouping

Due to updates in the T and M descriptors, adjustments of current staging categories and creation of new staging groups were designed for the eighth edition (Table 2) [98].

## 5. Lung Cancer Follow-Up and Response Evaluation

Surgery is typically the primary treatment modality for patients with localized disease. Post-treatment follow-up comprises imaging, physical examination, and laboratory tests combinations. There is no individual modality that can be sensitive, specific, and cost-effective at the same time; therefore, a mixed approach is required to detect tumor recurrence [46]. Proper imaging is a key model for response evaluation of lung cancer and is essential to identify progression of the disease during chemotherapy with cytotoxic agents, molecular targeting therapy, and blend therapy of both types [111].

## 6. World Health Organization (WHO) Criteria and Response Evaluation Criteria in Solid Tumors (RECIST)

Standardized tumor response criteria, such as WHO criteria and RECIST, are simple reasonable measures for response assessment through institutes [111]. These are mainly based on size, involving bidimensional tumor measurements. In the WHO criteria, these are the largest perpendicular dimensions in the axial direction, while RECIST uses the unidimensional diameter, which is the largest tumor dimension in the axial plane [111,112].

Though both WHO and RECIST criteria are broadly used in routine practice and clinical trials for assessing treatment response, they have a few limitations. Only maximum tumor diameters are employed for analysis without functional information [113]. Inter-reader variability has been reported for tumor size measurements, especially in irregular lesions, and thus may tend to misclassify tumor progression. In addition, the size measurements of lung cancer do not precisely discriminate a residual tumor from post-radiotherapy changes [46]. Additionally, the CT cannot reliably distinguish a histopathologic response (described as less than or equal to 10% viable tumor). After neoadjuvant chemotherapy, a discordance rate of 41% between histopathologic response and CT-based RECIST response classification was reported in patients with NSCLC [114].

FDG PET/CT is a potent tool in post-treatment response evaluation, and it predicts survival better than CT, as FDG PET/CT increases accuracy and prediction of disease progression since cellular metabolism changes arise more quickly than tumor size changes [115]. Criteria for assessing PET response in tumors have been presented to be a stronger predictor of pathologic response than anatomic response indicators, as WHO criteria, RECIST, and PET/CT detect the response by measuring the maximum SUV in the tumor [112].

The NCCN advised FDG PET/CT for proper staging and accurate radiation therapy (RT) planning for both NSCLC and SCLC, but the NCCN does not recommend the routine use of FDG PET/CT for assessment and follow-up treatment response [46].

Various studies demonstrated the benefit of post-treatment FDG PET/CT SUVmax to predicttreatment response and prognosis, usually achieved within 6 months after treatment completion [46,116]. Additionally, meta-analysis stated that the measured SUVmax in the tumor before and after RT was competent to predictpatient prognosis [117].

DWI functional assessment was of more value than CT in evaluating response after chemotherapy and/or radiotherapy in lung cancer [113]. DWI is comparable and could have a greater chance of predicting tumor response to therapy in NSCLC patients before chemoradiotherapy than FDG PET/CT. Both ADC and SUVmax values are accepted as radiologic examination-based biomarkers [118]. ADC measurements are a promising way to monitor early response or predict prognosis after NSCLC chemotherapy [119,120].

## 7. Advances in Lung Cancer Tumor Genomics and Precision Therapy

Recent discoveries in the characterization of tumor genomic abnormalities and effective precision-targeted therapy for certain genomic aberrations in lung cancer achieved innovative progress in lung cancer treatment and altered the treatment methodologies for lung cancer in the last decade. Precision therapy has been brought to the focus of lung cancer therapy [111,121].

Radiologists must keep up with the revolutionary world of genomic alterations and the rapidly evolving therapy with anticancer agents that target these genomic alterations in order to maintain effective communication with oncologists in multidisciplinary teams for the care of patients [111,121].

Somatic activating mutations of the tyrosine kinase domain of the epidermal growth factor receptor (EGFR) gene are a representative example of genomic alterations in NSCLC. They respondwell to EGFR tyrosine kinase inhibitors (EGFR-TKI), gefitinib, and erlotinib target therapy [122,123]. EGFR mutations are the most common in women with adenocarcinoma who have no history of cigarette smoking and are less common in elderly heavy-smoker patients [124].

The rearranged anaplastic lymphoma kinase (*ALK*) oncogene is another example of genomic alterations in NSCLC and adenocarcinoma. It is more common in women, young patients, non-smokers, or light smokers [125].

EGFR and *ALK* mutation testing is suggested for patients with NSCLC, adenocarcinoma, and large-cell carcinoma by the National Comprehensive Cancer Network Clinical Practice Guidelines in Oncology. If both are negative, chemotherapy is started. If positive for EGFR mutation, erlotinib target therapy is started; if positive for *ALK* mutation, crizotinib target therapy is started. Patients who suffer from squamous cell carcinoma are not recommended to undergo EGFR and *ALK* mutation testing, and should instead proceed to chemotherapy [126].

The precision therapy strategies for lung cancer are rapidly advancing, enabling the introduction of novel methods and genomic maps analyses into the clinical practice. The radiologist’s knowledge of these recent therapeutic plans and their relation to the pre- and post-treatment imaging become essential, especially among members of multidisciplinary lung cancer teams [121].

## 8. Conclusions

Updated knowledge of lung cancer genomic abnormalities, advanced imaging techniques, treatment regimens, and tumor response assessment helps the radiologists to continue as effective contributors to the customized care of lung cancer patients. Details of lung cancer classifications and different state-of-the-art screening techniques are presented in this review to serve as a source of learning and research.

## Figures and Tables

**Table 1:** Imaging appearance for TNM lung cancer staging in the eighth edition [98,101].

T Descriptor	
Tis (AIS)	Pure GGN is ≤3.0 cm.
T1mi	Less than or equal to 0.5 cm solid part in tumor total size ≤3.0 cm.
T1a	0.6–1.0 cm solid part in tumor size ≤3.0 cm.
	Pure GGN is greater than 3 cm.
	≤1 cm solid tumor.
T1b	1.1–2.0 cm solid part in tumor size less than or equal to 3.0 cm.
	Greater than 1.0–2.0 cm solid tumor.
T1c	2.1–3.0 cm solid part in tumor size less than or equal to 3.0 cm.
	Greater than 2.0–3.0 cm solid tumor.
T2a	3.1–4.0 cm.
	Invades main bronchus (no carinal involvement).
T2b	4.1–5.0 cm.
	Total/partial atelectasis, pneumonitis.
	Involves visceral pleura (PL1 or PL2).
T3	5.1–7.0 cm.
	Tumor nodule in the same lobe as the primary tumor.
	Directly invades any of the following: chest wall, parietal pleura (PL3), parietal pericardium, or phrenic nerve.
T4	Greater than 7.0 cm.
	Tumor nodule in different ipsilateral lobe than that of primary tumor.
	Directly invades any of the following: diaphragm, mediastinum, trachea, carina, great vessels, heart, recurrent laryngeal nerve, esophagus, or vertebral body.
**N Descriptor**	
N0	No LN metastasis.
N1	Metastasis to ipsilateral peribronchial, intrapulmonary, or hilar LNs.
N2	Metastasis to ipsilateral mediastinal or subcarinal LNs.
N3	Metastasis to ipsilateral or contralateral supraclavicular/scalene LNs.
	Metastasis to contralateral mediastinal, hilar LNs.
**M Descriptor**	
M0	No distant metastasis.
M1a	Malignant pleural effusion or pericardial effusion.
	Contralateral lung nodules/pleural nodules.
M1b	Single extrathoracic metastasis.
M1c	Multiple extrathoracic metastasis.

GGN, AIS, mi, PL1, PL2, PL3, and LN refer to ground-glass nodules, adenocarcinoma in situ, minimally invasive, tumor that reaches the elastic layer of visceral pleura without extending to its surface, tumor that reaches the surface of the visceral pleural, tumor that reaches the parietal pleura or chest wall, and lymph node, respectively [98,102,103].

**Table 2:** Overall stage grouping of TNM lung cancer in the eighth edition [98,101].

Stage	M	N	T
0	M0	N0	Tis
IA1	M0	N0	T1mi
	M0	N0	T1a
IA2	M0	N0	T1b
IA3	M0	N0	T1c
IB	M0	N0	T2a
IIA	M0	N0	T2b
IIB	M0	N1	T1a, b, c
	M0	N1	T2a, b
	M0	N0	T3
IIIA	M0	N2	T1a, b, c
	M0	N2	T2a, b
	M0	N1	T3
	M0	N0	T4
	M0	NI	T4
IIIB	M0	N3	T1a, b, c
	M0	N3	T2a, b
	M0	N2	T3
	M0	N2	T4
IIIC	M0	N3	T3
	M0	N3	T4
IVA	M1a	Any N	Any T
	M1b	Any N	Any T
IVB	M1c	Any N	Any T

## Data Availability

Not applicable.
